# Predictors Of Post-Operative Negative Outcomes in Patients Undergoing Transcatheter Aortic Valve Implantation

**DOI:** 10.12968/bjca.2022.0061

**Published:** 2022-08-30

## Abstract

With the advent of Transcatheter Aortic Valve Implantation (TAVI), populations who were previously not fit enough for heart valve surgery are now able to have a valve replacement without placing these higher risk individuals through major surgery. Complication rates following TAVI have reduced over the last few years, but recent studies suggest that the incidence of several complications remains high. Avoiding complications is key to reducing costs associated with TAVI, which is important given that the procedure is already more expensive than other treatment options. An emerging strategy for patients undergoing TAVI is to identify pre-operative factors predictive of post-operative adverse outcomes. This commentary summarises three systematic reviews exploring moderating factors for adverse events after TAVI surgery, with the aim of identifying statistically and clinically significant factors associated with poor surgical outcomes and contextualise these factors within clinical practice.

## Introduction

Aortic stenosis is the most common valvular lesion in the elderly, with epidemiological studies determining that one in 10 people will undergo the procedure before their eighties (Joseph et al. 2017). With the advent of Transcatheter Aortic Valve Implantation (TAVI), populations who were not fit enough for heart valve surgery are now able to have a valve replacement without placing higher risk individuals through major surgery (Howard et al. 2019). This procedure uses a catheter inserted either into an artery in the chest or upper leg (Nielsen 2012). The catheter is then used to insert the new folding aortic valve which is to be placed over the old aortic valve (Nielsen 2012). Research suggests that TAVI is increasingly seen as an acceptable alternative to conventional surgery in patients at high risk of mortality (Leon et al. 2010). Annual estimates suggest that more than 270,000 TAVI procedures are performed globally (Durko et al. 2018).

TAVI is described as a definitive therapy and is considered a key treatment to improve symptoms and survival for patients with symptomatic severe aortic stenosis (Falk et al. 2017). Three-year survival rates after TAVI in patients with symptomatic aortic stenosis is 87% (Díez 2013). This high survival rate is clinically and statistically significantly higher than for those who do not undergo surgery (Díez 2013). Complication rates following TAVI have reduced over the last few years but recent studies suggest that the incidence of several complications remains high (Eftychiou et al. 2021; Möllmann et al. 2015); for example, the incidence of stroke in the first month following TAVI has been reported at 4.9%, major adverse cardiovascular events at 4.3% and vascular complication as high as 7.2% (Eftychiou et al. 2021; Ricco et al. 2021).

Avoiding complications is key to reducing costs associated with TAVI, which is important given that the procedure is already more expensive than other treatment options (e.g., surgical aortic valve replacement) (Toggweiler 2014). Planning for TAVI procedures, particularly patient selection, has been found to lower rates of complications, resulting in shorter hospitalisation, lower costs and fewer deaths (Toggweiler 2014). An emerging strategy for patients undergoing TAVI is to identify preoperative factors predictive of post-operative adverse outcomes (Dautzenberg et al. 2021). Identifying predictors of hospital re-admission and mortality following TAVI can provide insight to help reduce complications and the costs associated with them (Dautzenberg et al. 2021; Ricco et al. 2021). Four recent systematic reviews in this area have assessed the association of a wide range of pre-operative factors and post-operative complications and mortality (Jiang et al. 2021; Li et al. 2021; Ullah et al. 2021; van Mourik et al. 2020).

## Aim of commentary

The aim of this commentary is to:

1. Critically appraise the methods used within the systematic reviews by Jiang, 2021, Ullah, 2021, van Mourik, 2020 and Li, 2021.
2. Identify statistically and clinically significant factors associated with poor surgical outcomes and contextualise these factors within clinical practice.

## Methods of Jiang, 2021, Ullah, 2021, van Mourik, 2020 and Li, 2021 systematic reviews

The inclusion criteria for all four reviews were similar. Only studies which included patients who underwent TAVI and reported clinical outcomes such as hospital readmission, all-cause mortality, incidence of prosthetic valve endocarditis or incidence of pacemaker implantation were included (Jiang et al., 2021; Ullah et al., 2021; Li et al., 2021; Van Mourik et al., 2020). The four reviews all included randomised control trials (RCTs), with three reviews also including observational studies (Jiang et al., 2021; Ullah et al., 2021; Li et al., 2021). All four reviews assessed pre-operative predictive factors (e.g., age, sex, body mass index, medical conditions, etc.) which may be associated with post-operative clinical outcomes. Three reviews assessed procedural and post-operative factors (Jiang et al., 2021; Ullah et al., 2021; Li et al., 2021). Table 1 provides a full overview of the population, exposures, outcomes, and study types of each review.

Using a modified Joanna Briggs Institute critical appraisal tool (Aromataris et al. 2015), the reviews were largely deemed to be of good methodological quality. All four reviews clearly described the aims, inclusion criteria, search strategy, criteria for appraising studies and methods of data analysis. The full quality assessment and corresponding methodological process is summarised in Table 2 and expanded upon in the commentary section.

## Results

All four reviews identify a range of statistically and clinically significant moderating factors. Full details of the results can be found within Table 3. A breakdown of each individual review is given below with particular focus on clinical and statistically significant moderating factors.

### Jiang et al, (2021)

Following screening of 279 citations, eight studies were included in the systematic review and meta-analysis. All eight studies scored ≥7 on the Newcastle-Ottawa Scaling System, indicating “high quality”. The eight studies included a total of 68,805 participants of which 1,256 (1.83%) were diagnosed with prosthetic valve endocarditis following Transcatheter Aortic Valve Replacement (TAVR). When pooled, the in-hospital mortality of patients diagnosed with prosthetic valve endocarditis following TAVR was 22.3% (n= 280).

The pre-operative factors of a person’s sex and age were associated with a statistically significant effect on the risk of prosthetic valve endocarditis following TAVR, with males at higher risk (relative risk (RR) 1.53; 95% CI: 1.24, 1.90; P = 0.0001) and younger people at lower risk (RR 0.97; 95% CI: 0.95, 0.99; P= 0.0001). Substantial heterogeneity affected the analysis of both predictive factors (I^2^ = 57% and 76% respectively). The presence of chronic obstructive pulmonary disease (COPD), diabetes or chronic renal insufficiency were not found to have a statistically significant effect on the risk of complications after TAVR following prosthetic valve endocarditis.

The procedural factor of orotracheal intubation was shown to increase the risk of prosthetic valve endocarditis following TAVR (RR 1.65; 95% CI: 1.12, 2.43; P= 0.01), although the analysis was affected by substantial heterogeneity (I^2^ = 60%). In contrast, self-expandable valves (RR 0.74; 95% CI: 0.58, 0.95; P= 0.02) and the transfemoral approach (RR 0.85; 95% CI: 0.71, 1.02; P=0.08) were associated with a lower risk of prosthetic valve endocarditis following TAVR, although the effect of the transfemoral approach was marginally insignificant.

The post-operative factors of new pacemaker implantation (RR 1.46; 95% CI: 1.14, 1.87; P= 0.003) and residual aortic regurgitation (RR 1.62; 95% CI: 1.01, 2.61; P=0.05) were associated with increased risk of prosthetic valve endocarditis following TAVR. The procedural factor of aortic regurgitation and the post-operative factors of vascular injury and bleeding complications appeared not significantly alter the risk of prosthetic valve endocarditis following TAVR

### Ullah et al, (2021)

After duplicate removal 2,009 citations were screened, with 75 observational studies and three RCTs included in the systematic review. All three RCTs (Cochrane Risk of Bias 2) and 73 observational studies (Newcastle-Ottawa Scaling System score ≥7) were classified as being of high methodological quality. The majority of the studies were undertaken within the United States and Europe. A total of 33,261 patients undergoing a TAVR were included in the review, with a mean age of 81 years old. The number of patients requiring a permanent pacemaker implantation ranged from 0.16% to 51.1%.

When pooled through a random effects meta-analysis, the odds of post TAVR permanent pacemaker implantation were significantly increased statistically (p<0.05) if the person had the following pre-operative characteristics: male (odds ratio (OR): 1.16; 95% CI: 1.04, 1.28); left anterior hemiblock (OR: 1.43; 95% CI: 1.09, 1.86); right bundle-branch block (OR: 2.48; 95% CI: 2.17, 2.83); bifascicular block (OR: 2.59; 95% CI: 1.52, 4.42); baseline Mobitz type 1 (OR: 3.13; 95% CI: 1.64, 5.93) or type 2 (OR: 3.89; 95% CI: 2.54, 5.95); intraprocedural atrioventricular block (OR: 4.17; 95% CI: 2.69, 6.46); and, left posterior fascicular hemiblock (OR: 3.34; 95% CI: 1.10, 11.13). Heterogeneity was thought to have a limited effect on these analyses (I^2^ <25%). A person’s age (>80 years) and whether they had first-degree heart block, atrial fibrillation, bundle branch block, severe pulmonary hypertension, moderate/severe mitral regurgitation, unspecified heart failure and heart failure with preserved ejection fraction had limited effect on the odds of post-TAVR permanent pacemaker implantation

Procedural factors also led to a statistically significant (p<0.05) increase in the odds of post TAVR permanent pacemaker implantation, including the use of: 29mm prosthesis (29mm vs. 23mm OR: 1.49; 95% CI: 1.06, 2.08); LOTUS aortic prosthesis (LOTUS vs. MCRS OR: 1.61; 95% CI: 1.23, 2.1); mechanically expandable prosthesis (mechanically expandable vs self-expanding OR: 1.44; 95% C:1.18, 1.76); and, self-expanding prosthesis (self-expanding vs balloon expandable OR: 1.93; 95% CI: 1.42, 2.6). Heterogeneity was not routinely reported.

### Van Mourik et al, (2020)

A total of 1,104 citations were assessed for selection after duplicate removal, of which 49 observational studies were included in the systematic review. When combined through meta-analysis, it was evident that if people had chronic lung disease (hazard ratio (HR) 1.57; 95% CI: 1.45, 1.71; p=not reported (NR)), chronic kidney disease (HR: 1.96; 95% CI: 1.68, 2.3; P=NR) or hypalbuminaemia (HR: 1.77; 95% CI: 1.38, 2.26; P=NR), there was a statistically and clinically significant increase in the risk of one-year mortality after TAVI (I^2^ <29%). Although the pre-operative characteristics of a person being underweight (BMI>20kgm^2^) (HR: 1.49; 95% CI: 1.1, 2.03; P=NR), having a low frailty score (HR: 2.16; 95% CI: 1.56, 3.00; P=NR) or gait speed under 6 seconds on a 5 metre walking test (HR: 13.35; 95% CI: 1.75, 101.69; P=NR) were identified as statistically and clinically significant factors in determining one-year mortality after TAVI, the meta-analyses were affected by substantial heterogeneity and should be interpreted cautiously (I^2^ > 86%). The remaining factors of anaemia and independence on activities of daily living prior to the procedure were found not to have a statistically significant effect on the risk of one-year mortality after TAVI.

### Li et al, (2021)

Ten observational studies were included from the 690 citations screened, with all assessed to be of good methodological quality (Newcastle-Ottawa Scaling System >7). The studies included 52,702 patients with a mean age of 82.8 years. Eight studies reported the incidence of early readmission after TAVI, which when pooled resulted in an overall event rate of 0.15 (95% CI: 0.11, 0.18).

A statistically significant increased risk of readmission within 30 days was associated with a history of heart failure (OR 1.14; 95% CI: 1.05, 1.24; P=NR), life-threatening or major bleeding during the procedure (OR 1.39; 95% CI: 1.2, 1.6; P=NR) and post-operative implantation of a new permanent pacemaker (OR 1.32; 95% CI: 1.16, 1.5; P=NR). Transfemoral (TF) access as part of the procedure appeared to have a statistically significant benefit in reducing the chance of 30-day readmission (OR: 0.69; 95% CI: 0.56, 0.84; P=NR). A history of diabetes appeared not to be an important risk factor in determining readmission within 30 days.

The chance of post-operative readmission at one year was shown to increase significantly if a person had a history of New York Heart Association Classification 3 or 4 (OR 1.29; 95% CI: 1.05, 1.58; P=NR) and pre-operative mitral regurgitation (OR 1.32; 95% CI: 1.16, 1.5; P=NR). Major or life-threatening bleeding during the procedure (OR 1.32; 95% CI: 1.13, 1.4; P=NR), as well as post-procedure acute kidney injury (OR 1.79; 95% CI: 1.37, 2.34; P=NR) and paravalvular leak (moderate and severe) (OR 1.44; 95% CI: 1.17, 1.77; P=NR), also significantly increased the risk of post-operative readmission at one year. In contrast, patients who were female (OR 0.82; 95% CI: 0.69, 0.98; P=NR) and for those where the procedure used transfemoral access (OR 0.80; 95% CI: 0.68, 0.95; P=NR) there was a statistically significant lower odds of one-year readmission. Other factors, specifically chronic obstructive pulmonary disease, peripheral vascular disease and post-procedural left ventricular ejection fraction, were found to be non-significant risk factors for hospital readmission at one year. It is important to recognise that several of the analyses were affected by moderate heterogeneity (I^2^ range 40%-60%), which may require some caution in interpreting the results.

## Commentary

Using the Joanna Briggs Checklist for Systematic Reviews, all four reviews were deemed satisfactory with scores of six or greater out of nine items (Aromataris et al. 2015). The main concern was that all four reviews used less than adequate sources and resources to search for studies. All reviews limited searches by date, but it was not made evident why the limit for publication was applied. Three reviews failed to provide a search strategy and searched less than four databases. These issues may have led to relevant studies being missed. Other concerns of methodological quality related to the uncertainty of whether critical appraisal and data extraction were conducted independently by two reviewers, and that no assessment of publication bias was reported (in two of the four reviews) (Tricco et al. 2022). Despite these concerns it was deemed that these reviews provide an accurate and comprehensive summary of the evidence available that address the question of interest.

The findings from these reviews suggest that there are multiple factors which can be identified pre-operatively, procedure-related and after TAVI which may be associated with post-operative negative outcomes (permanent pacemaker implantation, endocarditis mortality and readmission) (Jiang et al. 2021; Li et al. 2021; Ullah et al. 2021; van Mourik et al. 2020). As suggested within the NICE guidelines for TAVI for aortic stenosis, patient selection should be undertaken by a multidisciplinary team where the risks associated with the procedure are assessed and the most suitable procedure is selected (National Institute for health and care excellence 2017). When carrying out the patient selection process for TAVI, it is important to be aware that the following baseline characteristics are associated with poor post-operative outcomes: older age, males, chronic renal failure, chronic lung disease, obstructive pulmonary disease, chronic kidney disease, clinical heart failure, mitral regurgitation, hypoalbuminaemia, anaemic and a score of >3 on the New York Heart Association Classification (Ullah et al. 2021).

Alongside these baseline characteristics, standard post-operative assessments of electrocardiography and frailty (Otto et al. 2021) may help to identify key risk factors associated with post-operative negative outcomes (Ullah et al. 2021; van Mourik et al. 2020). When undertaking the pre-operative electrocardiography, the specific conduction abnormalities of intraprocedural atrioventricular block, Mobitz type-2 second-degree, left posterior fascicular hemiblock, Mobitz type-1 second-degree, bifascicular block, right bundle-branch block and left anterior fascicular hemiblock should be focused upon as these conduction abnormalities are associated with higher odds of permanent pacemaker implantation (Ullah et al. 2021). Similarly, the specific frailty factors of patients having a slow gait speed, reduced activities of daily living, low frailty score and being underweight are associated with one-year mortality after TAVI. Both these ECG and frailty factors should feed into the overall assessment of risk and procedural selection.

When selecting the specific approach for TAVI, each individual procedural factor should be risk assessed (Khan et al. 2019; Otto et al. 2021) for patients who it is deemed that a 29mm prosthesis, LOTUS valve or mechanically expandable prosthesis are at higher risk of post-operative negative outcomes. On the other hand, those who are planned to undergo TAVI using the transfemoral approach are at less risk of developing endocarditis after TAVI. After surgery, patients should be regularly monitored for the presence of residual aortic regurgitation, paravalvular leak and acute kidney injury as these factors have been identified to be associated with further negative postoperative outcomes. Using the factors identified in this commentary could help to facilitate better disease care pathway management. This management can take form in numerous ways. Cardiac rehabilitation is highly recommended for post-myocardial infarction, however to date there are no major guidelines that recommend it post-TAVI (23). Preliminary data is beginning to support its usefulness in reducing mortality (23). Alternatively, a rehabilitation program aimed at identifying high-risk individuals and employing optimization strategies through improving physical capacity, nutritional status and psychological readiness could improve post-operative outcomes (23).

Future research in this area could use the factors identified in this commentary to develop a preoperative and procedural selection risk score, which could be used to identify patients who are high risk. Most of these factors, as highlighted above, are commonly collected as standard preassessment strategies.

## CPD reflective questions

- What advice can be given to patients about predictive factors of post-operative negative outcomes associated with Transcatheter Aortic Valve Implantation?
- What are the key limitations of the evidence provided by the systematic reviews of this commentary?
- What factors are important when assessing for increased risk of mortality and hospital readmission for patients undergoing Transcatheter Aortic Valve Implantation?

## Figures and Tables

**Table 1:** Study characteristics of each review.

	Population	Exposure	Clinical outcome	Study type	Factors assessed (preoperative predictors)
Jiang, 2021 (Predictor for patients with prosthetic valve endocarditis (PVE) after TAVR)	No restrictions regarding participants, but review included cohort studies of patients with PVE and patients without PVE	Underwent Transcathe ter Aortic Valve Implantati on	Incidence of prosthetic valve endocarditis after TAVR	Studies illustrating the incidence and risk factors of PVE after TAVR and cohort studies including patients with PVE and patients without PVE (RCT’s, cohort or observationa l studies)	Age. Male sex. Diabetes. Chronic obstructive pulmonary disease (COPD) Chronic renal insufficiency
Ullah, 2021 (Predictors of Permanent Pacemaker Implantation in patients undergoing TAVR)	Patients undergoing TAVR	Underwent Transcathe ter Aortic Valve Implantati on	Permanent Pacemaker Implantation (PPI)	Randomized control trials and observationa l studies	Type-1 second-degree heart block Type-2 second-degree heart block Left anterior fascicular hemiblock Bi-fascicular block Right bundle-branch block Intraprocedural atrioventricular block Age First-degree heart block Atrial fibrillation Left posterior fascicular hemiblock Left bundle branch block Severe pulmonary hypertension Mitral regurgitation Unspecified heart failure Heart failure with preserved ejection fraction
**Van Mourik, 2020** (Preoperative frailty parameters as predictors for outcomes after transcatheter aortic valve implantation)	No restrictions as regards study participants, vascular access route, percutaneous valve type or other TAVI procedural characteristics	Underwent Transcathe ter Aortic Valve Implantati on	All-cause 1year mortality	Only randomised control trials	Chronic lung disease Chronic kidney disease Underweight (Body Mass Index >20kgm^2^) Hypoalbuminaemia Frailty Anaemia Gait speed Activities of daily living (ADL) independence
**Li et al, 2021** (Causes and predictors of readmission after transcatheter aortic valve implantation)	Patients undergoing TAVI	Underwent Transcathe ter Aortic Valve Implantati on	Causes and predictors of readmission after TAVI at short-term and mid-term follow-up	Randomized control trials and observationa l studies	Heart failure New permanent pacemaker implantation (PPM) Diabetes Bleeding Predictors of readmission within 1 year Paravalvular leak Acute kidney injury Chronic obstructive pulmonary disease Peripheral Vascular disease New York Heart Association Classification >= 3 Mitral regurgitation Bleeding Female Transfemoral

Key: TAVR-Transcatheter Aortic Valve Implantation, PVE - Predictor for patients with prosthetic valve endocarditis, TAVI -Transcatheter Aortic Valve Implantation, RCT – Random controlled trial, ADL – Activities of daily living, PPM - New permanent pacemaker implantation, COPD - Chronic obstructive pulmonary disease.

**Table 2:** Quality assessment and methods (Aromataris et al. 2015).

Criteria	Jiang et al, (2021)	Ullah et al, (2021)	Van Mourik et al, (2020)	Li et al, (2021)
1. Is the review question clearly and explicitly stated?	Yes - To determine pooled, final incidence and mortality of prosthetic valve endocarditis in this specific transcatheter aortic valve replacement population, and identify perioperative parameters that best discriminated between transcatheter aortic valve replacement patients with and without prosthetic valve endocarditis.	Yes – The aim was to identify various cardiac and noncardiac predictors that lead to permanent pacemaker implantation following transcatheter aortic valve replacement and gauge the risk of conduction abnormalities based on the type of prosthesis and access site used in transcatheter aortic valve replacement.	Yes - To find and pool frailty characteristics as predictors for 1year mortality after TAVI.	Yes – The aim was to investigate the causes and predictors of readmission after TAVI at short-term and mid-term follow-up.
2. Were the inclusion criteria appropriate for the review question?	Yes - see Table 1.	Yes - see Table 1.	Yes - see Table 1.	Yes - see Table 1.
3. Was the search strategy appropriate?	Yes-Full description of the search strategy. Relevant key terms and MeSH terms used.	Yes-Full description of the search strategy. Relevant key terms and MeSH terms used.	Yes-Full description of search strategy with example, and relevant key and MeSH terms.	Yes-Full description of search and relevant key terms but only three databases were searched.
4. Were the sources and resources used to search for studies adequate?	Unclear - It is indicated that studies were included up to February 2020, but no specific start date for the search strategy was reported.	Unclear - It is indicated that studies were included up to April 2021, but no specific start date for the search strategy was reported.	No – The review limited searches up to April 2018 and only searched 2 databases. They did include a search strategy.	No – The review limited searches 2002-2018 and only three databases were searched (did not include MEDLINE).
5. Were the criteria for appraising studies appropriate?	Yes – Assessment of bias was undertaken using Newcastle-Ottawa scale.	Yes - The risk of bias-2 (RoB-2) and the NewcastleOttawa Scale for assessing nonrandomized studies were used	Yes – Assessment was conducted using the Newcastle-Ottawa scale.	Yes – Assessment was conducted using the Newcastle-Ottawa scale.
6. Was critical appraisal conducted by two or more reviewers independently?	No - Exact process stated.	No - Exact process stated.	Yes - Assessed for quality by two reviewers and marked as ‘high’ or ‘low’ quality, based on full-text review.	Unclear - Process of critical appraisal not reported.
7. Were there methods to minimize errors in data extraction?	No - Exact process of data extraction not described.	Yes - Data was extracted by 9 authors independently. It is unclear how this was undertaken.	No - Exact process of data extraction not described.	Yes - Two authors (LYM and MFY) extracted the data independently.
8. Were the methods used to combine studies appropriate?	Yes –A random effects metaanalysis was carried out for estimates of risk, odds, and hazard ratios. The I^2^ index was used to assess heterogeneity.	Yes – A random effects metaanalysis was undertaken using both random (DerSimonian and Laird) and fixed effect models (Mantel Haenszel). Results are based upon random effects model. The I^2^ index was used to assess heterogeneity.	Yes - A random effects metaanalysis was carried out for estimates of hazard ratios. The I^2^ index was used to assess heterogeneity.	Yes - The DerSimonian and Laird random effects model was used to pool estimates of proportions of readmission with the STATA software command MetaProp. The I^2^ index was used to assess heterogeneity.
9. Was the likelihood of publication bias assessed?	Yes – Was carried out but incorrectly as there were less than 10 studies.	Yes – Using a visual inspection of a funnel plot.	No – Assessment of publication bias was not reported.	No – Assessment of publication bias was not reported.
Total criteria achieved/	6/9	7/9	6/9	6/9

**Table 3:** Full results of Jiang 2021, Ullah 2021, Van Mourik 2020 and Li 2020.

Study	Factor	Outcome	Number of studies	OR/RR/HR (95% CI)	Statistics *P* =	Heterogeneity I^2^, P=	Quality/Risk of bias of evidence
**Jiang et al, (2021)**	Younger Age (baseline)	Incidence of prosthetic valve endocarditis after TAVR	n= 7	RR: 0.97, (95% CI 0.95 - 0.99)	P= 0.007	I^2^ = 76%, P= 0.0003	Out of the 8 observational studies included all 8 studies Scored >7 on the NewcastleOttawa Scaling
	Male sex (baseline)		n= 8	RR: 1.53, (95% CI 1.24 - 1.90)	P= 0.0001	I^2^ = 57%, P= 0.02	
	Diabetes (baseline)		n= 8	RR: 1.08, (95% CI 0.86-1.35)	P= 0.51	I^2^ = 50%, P= 0.06	
	COPD (baseline)		n= 8	RR: 1.0, (95% CI 0.83 - 1.28)	P= 0.80	I^2^ = 49%, P=0.06	
	Chronic renal failure (baseline)		n= 8	RR: 0.95, (95% CI 0.81 - 1.11)	P= 0.49	I^2^ = 67%, P= 0.003	
	Aortic regurgitation (>moderate) (procedural)		n= 4	RR: 1.04 (95% CI 0.89, 1.21)	P=0.6	I^2^ = 0%, P= 0.6	
	Self-expandable valve (procedural)		n= 4	RR: 0.74 (95% CI 0.58, 0.95)	P= 0.02	I^2^ = 0%, P= 0.51	
	Orotracheal intubation (procedural)		n= 3	RR: 1.65 (95% CI 1.12, 2.43)	P=0.01	I^2^ = 60%, P= 0.08	
	Transfemoral approach (procedural)		n= 6	RR: 0.85 (95% CI 0.71, 1.02)	P=0.08	I^2^ = 0%, P= 0.82	
	New pacemaker implantation (postoperative)		n= 6	RR: 1.46 (95% CI 1.14, 1.87)	P=0.003	I^2^ = 0%, P= 0.64	
	Residual aortic regurgitation (postoperative)		n= 4	RR: 1.62 (95% CI 1.01, 2.61)	P=0.05	I^2^ = 43%, P= 0.15	
	Vascular injury (postoperative)		n= 6	RR: 1.20 (95% CI 0.70, 2.06)	P=0.51	I^2^ = 54%, P= 0.05	
	Bleeding complications (post-operative)		n= 4	RR: 1.34 (95% CI 0.75, 2.40)	P=0.32	I^2^ = 57%, P= 0.07	
**Ullah et al (2021)**	Male (baseline)	Permanent pacemaker Implantation	n=31	OR: 1.16, (95% CI, 1.04–1.28)	P= <0.05.	I^2^ = 0%, P=0.92	Out of the 75 observational studies included 73 studies scored >7 on the NewcastleOttawa Scaling System. The three random controlled trials were deemed to be of “high quality”.
	Electrocardiographic conduction abnormalities, Mobitz type-1 second-degree heart block (baseline)		n=3	OR: 3.13, (95% CI, 1.64-5.93)	P= <0.05.	I^2^ =0%, P=0.56	
	Electrocardiographic conduction abnormal, Mobitz type-2 second-degree heart block (baseline)		n=2	OR: 3.89, (95% CI, 2.54–5.95)	P= <0.05.	N/R	
	Left anterior fascicular hemiblock (baseline)		n=9	OR: 1.43, (95% CI, 1.09–1.86)	P= <0.05.	I^2^ = 0%, P=0.96	
	Bifascicular block(baseline)		n=4	OR: 2.59, 95% CI, 1.52–4.42)	P= <0.05.	I^2^ =24.5, P=0.39	
	Right bundle-branch block (baseline)		n=29	OR: 2.48, (95% CI, 2.17–2.83)	P= <0.05.	I^2^=14.3, P=0.59	
	Intraprocedural atrioventricular block (baseline)		n=3	OR: 4.17, (95% CI, 2.69–6.46)	P= <0.05.	I^2^ =0%, P=0.39	
	Left posterior fascicular hemiblock (baseline)		n=4	OR: 3.34, (95% CI, 1.1–11.13)	P= <0.05.	I^2^ =0%, P=0.81	
	In patients age>80 (baseline)		n=5	OR: 1.19, (95% CI, 0.95–1.49)	P= >0.05.	I^2^ = 0%, P=0.95	
	First-degree heart block (baseline)		n=16	OR: 1.09, (95% CI, 0.05–2.37)	P= >0.05.	I^2^ = 82.43, P=0.00	
	Atrial fibrillation (baseline)		n=31	OR: 1.05, (95% CI, 90.93–1.20)	P= >0.05.	I^2^ = 0%, P=0.16	
	Bundle branch block (baseline)		n=29	OR: 1.06, (95% CI, 0.87–1.29)	P= >0.05.	I^2^ =23.9%, P=0.27	
	Severe pulmonary hypertension (baseline)		n=3	OR: 1.78, (95% CI, 0.82–3.89)	P= >0.05.	N/R	
	Moderate/severe mitral regurgitation (baseline)		N/R	OR, 3.3; 95% CI, 0.59–18.32	P= >0.05.	N/R	
	Unspecified heart failure (baseline)		n=5	OR, 1.06; 95% CI, 0.72–1.55	P= >0.05.	I^2^=11.5%, P=0.24	
	Heart failure with preserved ejection fraction (baseline)		n=4	OR: 1.01 (95% CI 0.51–2.01)	P= >0.05.	I^2^ = 0%, P=0.28	
	29mm vs. 23mm prosthesis (procedural)		n=9	OR: 1.49 (95% CI, 1.06–2.08)	P= <0.05.	N/R	
	23mm vs. 26mm prosthesis (procedural)		n=2	OR: 1.12 (95% CI, 0.62-2.03)	P= >0.05.	N/R	
	IV septuminterventricular Septum >22mm vs. <22mm (procedural)		n=1	OR:1.65 (95% CI, 0.55-4.93)	P= >0.05	N/R	
	IV septuminterventricular Septum >11mm vs.<11mm (procedural)		n=1	OR: 1.71 (95% CI, 0.17-17.41)	P= >0.05	N/R	
	Left ventricular outflow tract (procedural)		n=1	OR: 1.65 (95% CI, 0.55-4.93)	P= >0.05	N/R	
	LOTUS vs. EvolutR (procedural)		n=4	OR: 1.44 (95% CI, 0.94-2.20)	P= >0.05	N/R	
	LOTUS vs. ESV (procedural)		N=1	OR: 2.80 (95% CI, 0.76-10.32)	P= >0.05	N/R	
	LOTUS vs. MCRS (procedural)		N=2	OR: 1.61 (95% CI, 1.23-2.1)	P= <0.05.	N/R	
	Mechanically expandable vs self-expanding (procedural)		N=5	OR, 1.44; (95% CI, 1.18–1.76)	P= <0.05	I^2^ = 0%, P=0.85	
	Self-expanding vs balloon expandable vs (procedural)		N=17	OR: 1.93, 95% CI, 1.42–2.6.	P= <0.05	I^2^ = 57%, P=0.00	
**Van Mourik et al (2020)**	Chronic lung disease (baseline)	1 year mortality after TAVI	N=27	HR: 1.57, (95% CI 1.45-1.71)	Not Reported	I^2^=27% p=0.1	14/29 studies are low quality [NOS]
	Chronic kidney disease (eGFR <30ml/min) (baseline)		N=8	HR: 1.96, (95% CI 1.68-2.3)		I^2^=7% p=0.38	2/8 studies are low quality [NOS]
	Underweight (BMI>20kgm^2^) (baseline)		N=7	HR: 1.49, (95% CI 1.1-2.03)		I^2^=86% p=<0.01	4/7 studies are low quality [NOS]
	Hypoalbuminaemia (<3.5g /dl or <4g/dl) (baseline)		N=5	HR: 1.77, (95% CI 1.38-2.26)		I^2^=28% P=0.24	2/5 studies are low quality [NOS]
	Low frailty score (baseline)		N=9	HR: 2.16, (95% CI 1.56-3.00)		I^2^=86% P<0.01	4/9 studies are low quality [NOS]
	Anaemia (baseline)		N=5	HR: 2.09, (95% CI 0.93-4.66)		I^2^=95% P<0.01	2/5 studies are low quality [NOS]
	Gait speed (<6s on 5-m walking test) (baseline)		N=3	HR: 13.35, (95% CI 1.75-101.69)		I^2^ 94% P<0.01	1/3 studies are low quality [NOS]
	ADL independence (Katz activities of daily living score of 1 or more deficit) (baseline)		N=2	HR: 5.17, (95% CI 0.77-34.57)		I^2^=93% P<0.01	1/2 studies are low quality [NOS]
**Li et al, (2021)**	New permanent pacemaker implantation (PPM) (post-operative)	30 day readmission	N=4	OR: 1.32, (95% CI 1.16-1.5)	Not reported	I^2^ = 36.8%	Out of the 10 observational studies included 73 studies scored >7 on the NewcastleOttawa Scaling System.
	History of Diabetes Mellitus (baseline)		N=4	OR: 1.09, (95% CI 0.9-1.32)		I^2^ = 81.5%	1/2 studies are low quality [NOS]
	Major bleeding or lifethreatening bleeding (procedural)		N=4	OR: 1.39, (95% CI 1.2-1.6)		I^2^ = 60%	Out of the 10 observational studies included 73 studies scored >7 on the NewcastleOttawa Scaling System.
	History of Clinical heart failure (baseline)		N=4	OR: 1.14, (95% CI 1.05-1.24)		I^2^ = 0%	
	Transfemoral (TF) access (procedural)		N=5	OR: 0.69, (95% CI: 0.56–0.84)		I^2^ = 71.7%	
	Paravalvular leak (moderate and severe) (post-operative)	1 year readmission	N=3	OR: 1.44, (95% CI 1.17-1.77)		I^2^ = 8.9%	
	Acute Kidney injury (AKI) (post-operative)		N=3	OR: 1.79, (95% CI 1.37-2.34)		I^2^ = 0%	
	History of Chronic obstructive pulmonary disease (COPD) (baseline)		N=4	OR: 1.09, (95% CI 0.79-1.5)		N/R	
	History of Peripheral Vascular disease (baseline)		N=3	OR: 1.14, (95% CI 0.90-1.44)		N/R	
	History of New York Heart Association Classification >= 3 (baseline)		N=3	OR: 1.29, (95% CI 1.05-1.58)		I^2^ = 42.7%	
	History of Mitral Regurgitation (moderate and severe) (baseline)		N=5	OR: 1.32 (95% CI 1.16-1.5)		I^2^ = 36.8%	
	Major bleeding or lifethreatening bleeding (procedural)		N=4	OR: 1.32, (95% CI 1.13-1.4)		I^2^ = 51.9%	
	Female (baseline)		N=3	OR: 0.82, (95% CI 0.69–0.98)		N/R	
	Transfemoral (TF) access (procedural)		N=4	OR: 0.80, (95% CI 0.68–0.95)		N/R	
	Left ventricular ejection fraction post-TAVI (postoperative)		N=3	OR: 1.02, (95% CI 0.98–1.06)		N/R	

* OR= odds ratio, RR= risk ratio, HR= Hazard ratio, CI= confidence intervals, NOS= Newcastle-Ottawa Scale, TAVI= transcatheter aortic valve replacement, N/R= not reported

## Key Points

- Pre-operative factors of being male, chronic renal failure, chronic lung disease, obstructive pulmonary disease, chronic kidney disease, clinical heart failure, mitral regurgitation, hypoalbuminaemia, anaemic and a score of >3 on the New York Heart Association Classification are associated with poor post-operative outcomes.
- Procedural factors of 29mm prosthesis, LOTUS valve or mechanically expandable prosthesis are associated with an increased risk of post-operative negative outcomes.
- Post-operative factors of residual aortic regurgitation, paravalvular leak and acute kidney injury have been identified to be associated with negative post-operative outcomes.

## Commentary on

1. Jiang, W., Wu, W., Guo, R., Xie, M., Yim, W. Y., Wang, Y., & Hu, X. (2021). Predictors of Prosthetic Valve Endocarditis following Transcatheter Aortic Valve Replacement: A MetaAnalysis. The heart surgery forum, 24(1), E101-E107. https://doi.org/10.1532/hsf.3461

2. Ullah, W., Zahid, S., Zaidi, S. R., Sarvepalli, D., Haq, S., Roomi, S., Mukhtar, M., Khan, M. A., Gowda, S. N., Ruggiero, N., Vishnevsky, A., & Fischman, D. L. (2021). Predictors of Permanent Pacemaker Implantation in Patients Undergoing Transcatheter Aortic Valve Replacement - A Systematic Review and Meta-Analysis. Journal of the American Heart Association, 10(14), e020906. https://doi.org/10.1161/JAHA.121.020906

3. van Mourik, M. S., Velu, J. F., Lanting, V. R., Limpens, J., Bouma, B. J., Piek, J. J., Baan, J., Henriques, J., & Vis, M. M. (2020). Preoperative frailty parameters as predictors for outcomes after transcatheter aortic valve implantation: a systematic review and meta-analysis. Netherlands heart journal: monthly journal of the Netherlands Society of Cardiology and the Netherlands Heart Foundation, 28(5), 280-292.

4. Li, Y. M., Mei, F. Y., Yao, Y. J., Tsauo, J. Y., Peng, Y., & Chen, M. (2021). Causes and predictors of readmission after transcatheter aortic valve implantation: A meta-analysis and systematic review. Ursachen und Prädiktoren der Wiederaufnahme nach TranskatheterAortenklappenimplantation : Eine Metaanalyse und systematische Übersicht. Herz, 46(Suppl 1), 1-8. https://doi.org/10.1007/s00059-019-04870-6
